# Parental risk factors and anorectal malformations: systematic review and meta-analysis

**DOI:** 10.1186/1750-1172-6-25

**Published:** 2011-05-17

**Authors:** Nadine Zwink, Ekkehart Jenetzky, Hermann Brenner

**Affiliations:** 1 Division of Clinical Epidemiology and Aging Research, German Cancer Research Center, Heidelberg, Germany

**Keywords:** anorectal malformations, imperforate anus, anal atresia, birth defects, risk factors, pregnancy

## Abstract

**Background:**

Anorectal malformations (ARM) are rare forms of congenital uro-rectal anomalies with largely unknown causes. Besides genetic factors, prenatal exposures of the parents to nicotine, alcohol, caffeine, illicit drugs, occupational hazards, overweight/obesity and diabetes mellitus are suspected as environmental risk factors.

**Methods:**

Relevant studies published until August 2010 were identified through systematic search in PubMed, EMBASE, ISI Web of Knowledge and the Cochrane Library databases. Furthermore, related and cross-referencing publications were reviewed. Pooled odds ratios (95% confidence intervals) were determined to quantify associations of maternal and paternal smoking, maternal alcohol consumption, underweight (body mass index [BMI] < 18.5), overweight (BMI 25-29.9), obesity (BMI ≥30) and maternal diabetes mellitus with ARM using meta-analyses.

**Results:**

22 studies that reported on the association between prenatal environmental risk factors and infants born with ARM were included in this review. These were conducted in the United States of America (n = 12), Spain (n = 2), Sweden (n = 2), the Netherlands (n = 2), Japan (n = 1), France (n = 1), Germany (n = 1) and Hungary (n = 1). However, only few of these studies reported on the same risk factors. Studies were heterogeneous with respect to case numbers, control types and adjustment for covariates. Consistently increased risks were observed for paternal smoking and maternal overweight, obesity and diabetes, but not for maternal smoking and alcohol consumption. In meta-analyses, pooled odds ratios (95% confidence intervals) for paternal smoking, maternal overweight, obesity, pre-gestational and gestational diabetes were 1.53 (1.04-2.26), 1.25 (1.07-1.47), 1.64 (1.35-2.00), 4.51 (2.55-7.97) and 1.81 (1.23-2.65), respectively.

**Conclusion:**

Evidence on risk factors for ARM from epidemiological studies is still very limited. Nevertheless, the few available studies indicate paternal smoking and maternal overweight, obesity and diabetes to be associated with increased risks. Further, ideally large-scale multicentre and register-based studies are needed to clarify the role of key risk factors for the development of ARM.

## Introduction

In recent years, a number of studies have shown that prenatal exposures of the parents are associated with an increased risk for having a malformed child. However, only few studies exist regarding the association with anorectal malformations (ARM).

ARM are rare birth defects concerning anus and rectum. Approximately 1 in 2,500 to 1 in 5,000 new born babies are affected [1-3]. Different degrees of severity are distinguished, ranging from mild anal stenosis over anal atresia with or without fistula to persistent cloaca or even cloacal exstrophy [4]. Furthermore, ARM frequently manifest with other malformations. Approximately 64% of all ARM patients are affected and have one or more additional extra-anal anomalies [5]. Previous studies have shown that associated malformations are more frequent in "high" defects that are complex and difficult to manage with a poor functional prognosis than in "low" defects that are less complex and easily treated with an excellent functional prognosis. Associated malformations mainly include the genitourinary system (21-61% and more), spine and spinal cord (5-40%), the rest of the gastrointestinal tract (10-25%) and the heart (9-20%) [6]. Anorectal malformations affect several socioeconomic and ethnic groups [7-11]. Boys seem to be at a slightly higher risk than girls (1.3:1) [12]. It is assumed that the defects occur during the 4th to 8th week of fetal development [13-18]. Current knowledge about the causes, however, is still sparse. In addition to genetic factors, prenatal exposures of the parents to tobacco, alcohol, caffeine, illicit drugs, overweight/obesity, diabetes mellitus and occupational hazards are subject to ongoing debate as potential environmental risk factors, in particular because the few existing studies are based on retrospectively collected data from individual centers only.

We conducted a systematic review and meta-analysis of epidemiological studies to summarize current evidence on the relationship between parental risk factors and anorectal malformations and to identify knowledge deficits that need to be addressed in future research.

## Methods

### Identification of studies and study selection

A literature search was carried out to identify epidemiological studies assessing the association between seven prenatal exposures of parents that have been suggested to be environmental risk factors for anorectal malformations: smoking, alcohol, caffeine, illicit drugs, overweight/obesity, diabetes and occupational hazards. Relevant studies published in English were systematically searched in PubMed, EMBASE, ISI Web of Knowledge and the Cochrane Library databases by using various combinations of the following terms: (congenital malformation(s), congenital abnormality, congenital abnormalities, birth defect(s), anorectal malformation(s), anorectal atresia, anal atresia, imperforate anus) AND (smok*, nicotine, tobacco, cigarette*, alcohol*, drink*, caffeine*, coffee*, illicit drug(s), drug(s), overweight, obesity, adiposity, diabetes (mellitus), diabetes type 2, diabetes type 1, type 2 diabetes, type 1 diabetes, gestational diabetes, pre-gestational diabetes, pre-existing diabetes, occupational hazard(s), occupational risk(s), professional risk(s), job hazard(s), parental occupation, maternal occupation, paternal occupation). Duplicate articles were deleted. Each title and abstract was checked for relevance. The full text was reviewed if the abstract indicated that the article reported an association between ARM and one of the previously mentioned risk factors. Furthermore, the identified articles were reviewed for related articles and cross-referring publications.

### Inclusion criteria

Articles were included if they reported on associations of anorectal malformations with at least one of the previously mentioned environmental risk factors. When available, data of ARM infants with isolated anomalies (no additional major defects) were preferred to data of ARM infants with multiple defects. Articles were excluded if the reported number of ARM cases was less than two. ARM infants analysed only in a group with other anomalies like intestinal or tracheo-esophageal atresias were also excluded because of concern that associations of risk factors with these anomalies might be different from associations with ARM. Searches were restricted to English-language articles.

### Data extraction

Two reviewers independently assessed the articles and extracted the following key information in a standardized manner: first author, year, country, study design, characteristics of the study population, period of data acquisition, assessed risk factor(s) for ARM and the respective measures of odds ratio or prevalence ratio (see below), as well as covariates adjusted for in the analysis. Initial disagreements on classifications of study characteristics were resolved by discussion within the team of authors.

Associations between parental exposures and ARM are presented by odds ratios (OR) and their 95% confidence intervals (CI). Alternatively, reported prevalence ratios (PR) are shown. Unadjusted values were recalculated by the Review Manager Software, version 5.0.24 (The German Cochrane Centre, Freiburg, Germany) to validate the results. When measures of associations were not explicitly reported, they were derived from data provided in the articles.

### Meta-analyses

Meta-analyses were performed for risk factors for which results were available from at least two studies. Heterogeneity was assessed by the χ² and I² statistics. When the number of studies is low or when sample sizes are small, the power of the χ² test is low. The I² measure describes the proportion of total variation in effect estimates across studies that is due to heterogeneity rather than sampling error [19]. Fixed and random effects models were calculated by the R^© ^software, version 2.11.1 (The R Foundation for Statistical Computing, Vienna) using standard meta-analysis methods. The fixed effects model was used to estimate the variance of the summary odds ratio when study heterogeneity was low (I²≤ 25) and the random effects model when study heterogeneity was moderate to high (I²> 25) [20,21]. Indication of publication bias was assessed by Begg and Mazumdar rank correlation test [22] and Egger's test [23] (P < 0.1).

## Results

### Literature search result

**Figure 1:**
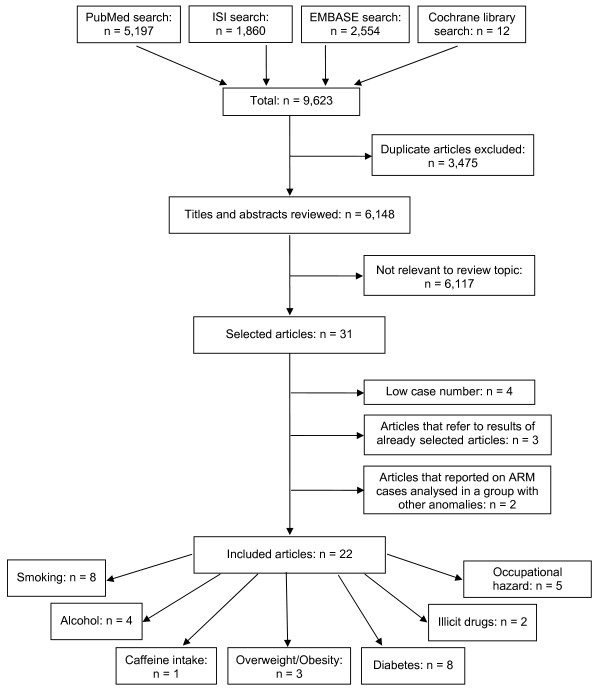
**Flow diagram of the literature search process**.

In total, 9,623 articles were found (figure 1). After removal of 3,475 duplicates, 6,148 titles and abstracts were reviewed. Thirty-one articles appeared to be potentially relevant for inclusion in the review. Of these, four articles were excluded because of too low case numbers (n < 2), three articles because they referred to results of already selected articles and further two articles because they reported on ARM cases analysed in a group with other anomalies. Finally, 22 articles were included in the review. Among the included studies, eight provided data on the association of ARM with prenatal exposures to smoking, four to alcohol consumption, one to caffeine intake, two to illicit drug use, three to overweight/obesity, eight to diabetes mellitus and five to occupational hazards.

### Studies included in this review

Details on the 22 studies, which were published from 1981 to June 2010, are shown in table 1. Studies were mainly conducted in the USA (n = 12). The remaining studies were conducted in Spain (n = 2), Sweden (n = 2), the Netherlands (n = 2), Japan (n = 1), France (n = 1), Germany (n = 1) and Hungary (n = 1). Recruitment was population-based in 16 studies and hospital-based in six studies. For data acquisition, nine studies relied on register-based data [1,7,24-30]. Data acquisition periods varied from one year [31] to 29 years [32].

**Table 1:** Case-control and cross-sectional studies reporting on the association of ARM and environmental risk factors

		Study population						
					
			No. participants					
							
Ref.	First author, year	Country	Cases	Controls	Age range	Setting, control type	Data acquisition (period)	Assessed risk factor(s)
[25]	Bánhidy, 2010	Hungary	231	38,151	< 19 - > 35	population-based, no birth defects	data from the Hungarian Case-Control Surveillance of Congenital Abnormalities (1980-1996)	diabetes
[26]	Blomberg, 2010^¥^	Sweden	401	1,049,181	< 20 - ≥45	population-based, all infants	data from the Swedish Medical Birth Registries^† ^(1995-2007)	overweight/obesity
[35]	Herdt-Losavio, 2010	USA	328	3,833	< 20 - ≥35	multistate population-based, no birth defects	data from the National Birth Defects Prevention Study (NBDPS) (1997-2003)	occupational hazard
[39]	van Rooij, 2010	Netherlands	85	650	≥35	hospital-based, no major birth defects	questionnaire (1996-2008^‡^)	smoking, alcohol, overweight/obesity, occupational hazard
[37]	Miller, 2009	USA	464, 216**^#^**	4,940	≤ 19 - ≥35	multistate population-based, no major birth defects	data from the National Birth Defects Prevention Study (NBDPS) (1997-2003)	smoking, alcohol, caffeine
[41]	van Gelder, 2009	USA	456-468	4,967	< 20 - ≥35	multistate population-based, no major birth defects	data from the National Birth Defects Prevention Study (NBDPS), collected by telephone interview (1997-2003)	illicit drugs of mothers (between one month before pregnancy and the end of the third month of pregnancy)
[34]	Correa, 2008	USA	230 200**^#^**	4,689	< 20 - ≥35	multistate population-based, no major birth defects	data from the National Birth Defects Prevention Study (NBDPS) (1997-2003)	diabetes
[1]	Forrester, 2007	USA	162	316,346	N.A.	state-wide population-based, all live births	data from the Hawaii Birth Defects Program (HBDP), collected through review of medical records (1986-2002)	illicit drugs of mothers (during pregnancy and 1 year after delivery)
[32]	Frías, 2007^¥^	USA	417^Δ^, 427^ΔΔ^	29,722^Δ^, 30,509^ΔΔ^	N.A.	hospital-based, other malformed infants	data from the Spanish Collaborative Study of Congenital Malformations (ECEMC) (1976-2005)	diabetes
[40]	Waller, 2007	USA	380, 77**^#^**	4,065	< 18 - ≥35	multistate population-based, no birth defects	data from the National Birth Defects Prevention Study (NBDPS) (1997-2002)	overweight/obesity
[7]	Correa, 2003	USA	56, 32**^#^**	3,029	< 20 - ≥30	population-based, no birth defects	data from the Metropolitan Atlanta Congenital Defects Program (MACDP) (1968-1980)	diabetes
[24]	Aberg, 2001	Sweden	15	600	N.A.	population-based, other malformed infants	data from the Swedish Medical Birth Registries^† ^(1987-1997)	diabetes
[31]	Honein, 2001	USA	564	6,160,942	< 30 - ≥30	population-based, all live births	US public-use natality data tapes (National Vital Statistics System, National Centre for Health Statistics) (1997-1998)	smoking
[36]	Martínez-Frías, 1998^¥^	Spain	227	19,377	N.A.	hospital-based, other malformed infants	data from the Spanish Collaborative Study of Congenital Malformations (ECEMC) (1976-1995)	diabetes
[38]	Stoll, 1997	France	108, 51**^#^**	108	F: mean age 26.9, M: mean age 29.9	hospital-based, no birth defects	interview (1979-1995)	smoking, alcohol, diabetes, X-ray examinations
[27]	Cornel, 1996	Netherlands	52	3,962	≤ 20 - ≥40	population-based, other malformed infants	data from the Northern Netherlands (NNL) (1981-1994)	smoking
[29]	Schnitzer, 1995	USA	70	2,279	F: < 20 - ≥40, M: < 20 - ≥45	population-based, no birth defects	data from the Metropolitan Atlanta Congenital Defects Program (MACDP) (1968-1980)	occupational hazard
[30]	Yuan, 1995	Japan	84, 49**^#^**	174	F: 29.1 ± 4.9, M: 32.1 ± 5.6	population-based, no birth defects	data from the Kanangawa Birth Defects Monitoring Program (KAMP) (1989-1994)	smoking, alcohol
[43]	Martínez-Frías, 1994^¥^	Spain	196	18,563	N.A.	hospital-based, other malformed infants	data from the Spanish Collaborative Study of Congenital Malformations (ECEMC) (1976-1992)	diabetes
[28]	Matte, 1993	USA	103	2,403	< 20 - > 35	population-based, no birth defects	data from the Metropolitan Atlanta Congenital Defects Program (MACDP) (1968-1980)	occupational hazard
[33]	Shiono, 1986	USA	14	578	N.A.	population-based, other malformed infants	data from the Kaiser-Permanente Birth Defects Study (1974-1977)	smoking
[42]	Angerpointer, 1981	Germany	78 78 78 78	210* 169** 75*** 53****	< 20 - > 40	hospital-based, other malformed infants	questionnaire (1970-1974)	smoking

# ARM infants with isolated (no additional major defects) anomaly

† The Swedish Medical Birth Registry, the Swedish Register of Birth Defects (previously called the Registry of Congenital Malformations) and the National Patient Register (previously called the Hospital Discharge Registry)

‡ Difference in case and control period: cases 1996-2008, controls 1996-2004

Δ ARM infants for the examination of maternal pre-gestational diabetes

ΔΔ ARM infants for the examination of maternal gestational diabetes

* Control group includes 41 infants with esophageal atresia, 41 with stenosis/atresia of the small and large bowel, 75 with Hirschsprung's disease, 28 with omphalocele and 25 with gastroschisis

** Control group includes 41 infants with esophageal atresia, 75 with Hirschsprung's disease, 28 with omphalocele and 25 with gastroschisis

*** Control group includes 75 infants with Hirschsprung's disease

**** Control group includes 28 infants with omphalocele and 25 with gastroschisis

¥ Cross-sectional study

M = male; F = female; BMI = body mass index; N.A. = not available

Case numbers ranged from 14 ARM cases [33] to 564 ARM cases [31]. Children with known chromosomal anomalies were excluded in nine studies [26,27,34-40]. Twelve studies used healthy newborns or infants with no major birth defects as control group [7,25,28-30,34,35,37-41] and seven studies used malformed infants with other anomalies than ARM [24,27,32,33,36,42,43]. Controls of the remaining three studies were all infants born in the same settings during the respective study period [1,26,31]. Only six studies examined ARM infants with isolated anomalies [7,30,34,37,38,40].

### Findings for the reviewed risk factors

Study results as well as the covariates adjusted for are shown in tables 2 to 8.

**Table 2:** Associations between periconceptional exposures to tobacco

			Maternal smoking		Paternal smoking		Smoking of both parents	
						
Ref.	First author, year	Exposure	OR [95% CI]	*P *value	OR [95% CI]	*P *value	OR [95% CI]	Adjustment/matching factors
[39]	van Rooij, 2010	Cigarette consumption before or during pregnancy	0.8 [0.5, 1.3]	0.61	1.8 [1.1, 2.9]^§^	0.01	-	-
[37]	Miller, 2009^§§^	Non-smoker not exposed to ETS Non-smoker exposed to ETS at home or work Non-smoker exposed to ETS at home and work Smoked < 0.5 pack/day Smoked ≥0.5 pack/day Any smoking	1.0 Reference 1.1 [0.8, 1.5] 1.4 [0.5, 4.0] 1.0 [0.5, 1.8] 1.2 [0.8, 1.7] 1.1 [0.8, 1.6]	-	-		-	None of the variables met the criteria for confounding by the author; therefore, only the unadjusted odds ratios were presented
[31]	Honein, 2001	Any smoking 1-5 cigarettes/day 6-10 cigarettes/day 11-20 cigarettes/day ≥21 cigarettes/day	PR: 1.19 [0.94, 1.50] PR: 0.95 [0.60, 1.50] PR: 1.38 [1.00, 1.90] PR: 1.19 [0.80, 1.79] PR: 0.94 [0.29, 2.98]	-	-		-	Adjusted for: maternal age, education and race/ethnicity
[38]	Stoll, 1997	Any smoking	0.98 [0.94, 1.02]	-	-		-	-
[27]	Cornel, 1996	Any smoking	2.24 [1.15, 4.16]	0.01	-		-	-
[30]	Yuan, 1995	Any smoking	-	-	1.14 [0.59, 2.18]	0.70	1.75 [0.63, 4.87]	Matched by: maternal age groups (5-years interval), sex, parity and season of birth
[33]	Shiono, 1986	Any smoking	0.41 [0.09, 1.87]	0.25	-		-	-
[42]	Angerpointer, 1981	Any smoking ≥5 cigarettes per day	0.95 [0.45, 1.99]* 0.99 [0.46, 2.14]^†^ 1.20 [0.47, 3.10]^‡^ 0.63 [0.25, 1.57]^#^ 2.30 [1.00, 5.31]* 2.92 [1.16, 7.37]^†^ 1.58 [0.51, 4.83]^#^	0.89 0.98 0.70 0.32 -	-		-	-

* Control group includes 41 infants with esophageal atresia, 41 with stenosis/atresia of the small and large bowel, 75 with Hirschsprung's disease, 28 with omphalocele and 25 with gastroschisis

† Control group includes 41 infants with esophageal atresia, 75 with Hirschsprung's disease, 28 with omphalocele and 25 with gastroschisis

‡ Control group includes 75 infants with Hirschsprung's disease

# Control group includes 28 infants with omphalocele and 25 with gastroschisis

§ Paternal cigarette consumption 3 months before conception

§§ Exposure during the month before pregnancy to the third month of pregnancy

ETS = environmental tobacco smoke; PR = prevalence ratio

### Cigarette consumption

Seven studies reported on the association between maternal smoking before or during pregnancy and infants born with an anorectal malformation (table 2). Any smoking during pregnancy was significantly associated with ARM only in the study by Cornel et al. [27] (OR, 2.24; 95% CI, 1.15-4.16; P = 0.01). In the study by Angerpointer et al. [42] different control groups were used. The comparison with one group of control infants with esophageal atresia, Hirschsprung's disease, omphalocele and gastroschisis showed a significant association between smoking at least five cigarettes per day and ARM (OR, 2.92; 95% CI, 1.16-7.37). A similar trend was observed when using another control group of infants with esophageal atresia, stenosis/atresia of the small and large bowel, Hirschsprung's disease, omphalocele and gastroschisis (OR, 2.30; 95% CI, 1.00-5.31). In contrast, no association at all was observed in analyses for any maternal smoking, regardless of the control group used. Honein et al. [31] observed a marginally increased risk for the maternal consumption of 6-10 cigarettes per day (PR, 1.38; 95% CI, 1.00-1.90). The remaining studies could not confirm an association. Only two reviewed studies examined the association with paternal tobacco consumption. A significant association was observed by van Rooij et al. [39] (OR, 1.8; 95% CI, 1.1-2.9; P = 0.01) whereas the study by Yuan et al. [30] could neither confirm this finding nor an association of ARM with smoking of both parents.

**Figure 2:**
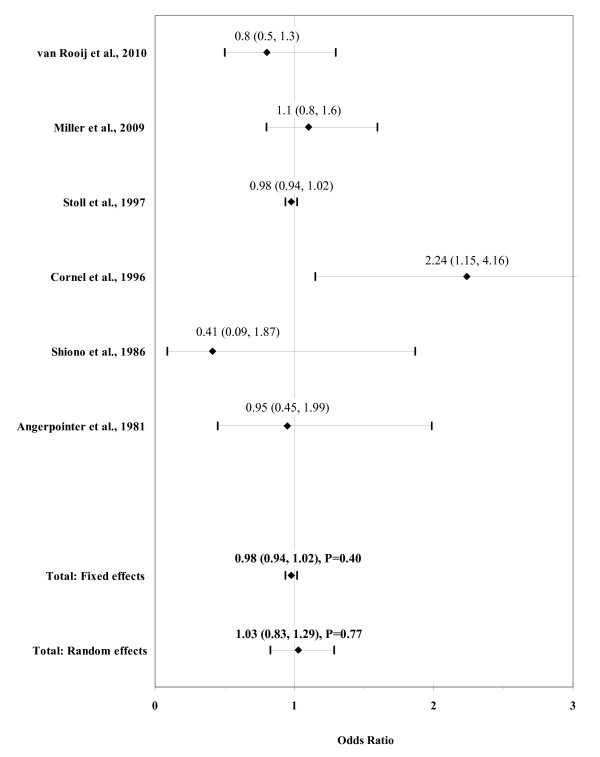
**Forest plot for maternal cigarette consumption**.

The result of the meta-analysis on the association between any maternal cigarette consumption and ARM infants is shown in figure 2. From the study by Angerpointer et al. [42] we used the OR calculated with the group of control infants with esophageal atresia, stenosis/atresia of the small and large bowel, Hirschsprung's disease, omphalocele and gastroschisis. The I² statistic indicated heterogeneity across studies (χ² = 8.72; P = 0.12; I² = 42.6%). The estimated heterogeneity variance was tau² = 0.0284. No significant association was observed in pooled analyses using the random effects model (OR, 1.03; 95% CI, 0.83-1.29; P = 0.77). There was no evidence of publication bias (Kendall's tau = -0.56, P = 0.57; Egger's t value = 0.32, P = 0.77).

**Figure 3:**
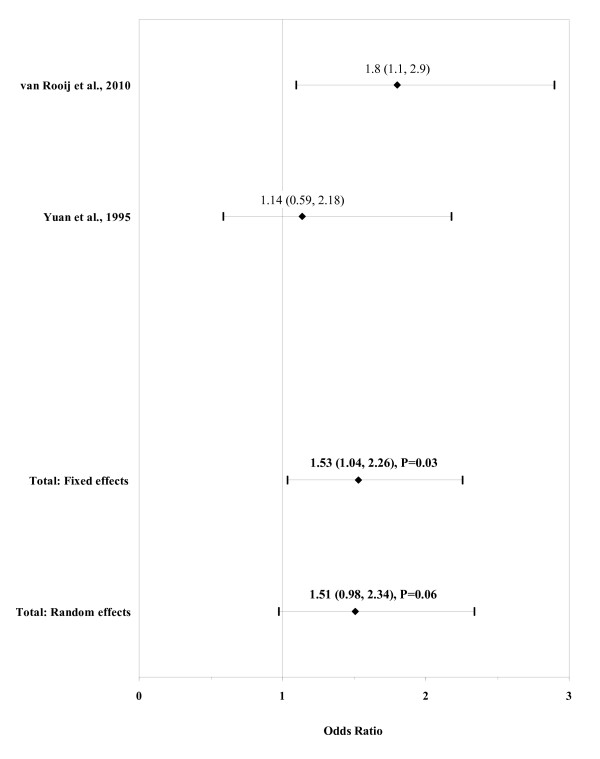
**Forest plot for paternal cigarette consumption**.

The result of the meta-analysis on the association between any paternal cigarette consumption and ARM infants is shown in figure 3. The I² statistic indicated low heterogeneity across the two studies (χ² = 1.21; P = 0.27; I² = 17.5%). In meta-analysis, a weak association was found for any paternal cigarette consumption using a fixed effects model (OR, 1.53; 95% CI, 1.04-2.26; P = 0.03).

### Alcohol consumption

Among four studies assessing maternal alcohol consumption, the association with ARM was significant only in the one by Yuan et al. [30] (OR, 4.77; 95% CI, 1.39-16.38) (table 3). The closer examination of alcohol quantity by Miller et al. [37] (no use, light use [≤ 1.5 drinks per day], heavy use [> 1.5 drinks per day] and binge drinking [≥5 drinks on at least one occasion]) did not show any indication of a possible dose-response relationship.

**Table 3:** Associations between periconceptional exposures to alcohol

		Maternal alcohol use			Paternal alcohol use			
				
Ref.	First author, year	Exposure	OR [95% CI]	*P *value	Exposure	OR [95% CI]	*P *value	Adjustment/matching factors
[39]	van Rooij, 2010	Alcohol consumption before or during pregnancy	1.0 [0.6, 1.5]	1.0	Alcohol consumption three months before conception	1.3 [0.7, 2.5]	0.33	-
[37]	Miller, 2009*	Non-drinker Average ≤ 1.5 drink/day Average > 1.5 drink/day ≥5 alcoholic drinks Drank any alcohol	1.0 Reference 1.0 [0.7, 1.5] 1.2 [0.9, 1.6] 0.9 [0.6, 1.6] 0.9 [0.7, 1.2]	-	-	-	-	None of the variables met the criteria for confounding by the author; therefore, only the unadjusted odds ratios were presented
[38]	Stoll, 1997	Any alcohol	1.25 [0.07, 21.04]	-	-	-	-	-
[30]	Yuan, 1995	Any alcohol	4.77 [1.39, 16.38]	-	-	-	-	Matched by: maternal age groups (5-years interval), sex, parity and season of birth

* Exposure during the month before pregnancy to the third month of pregnancy.

**Figure 4:**
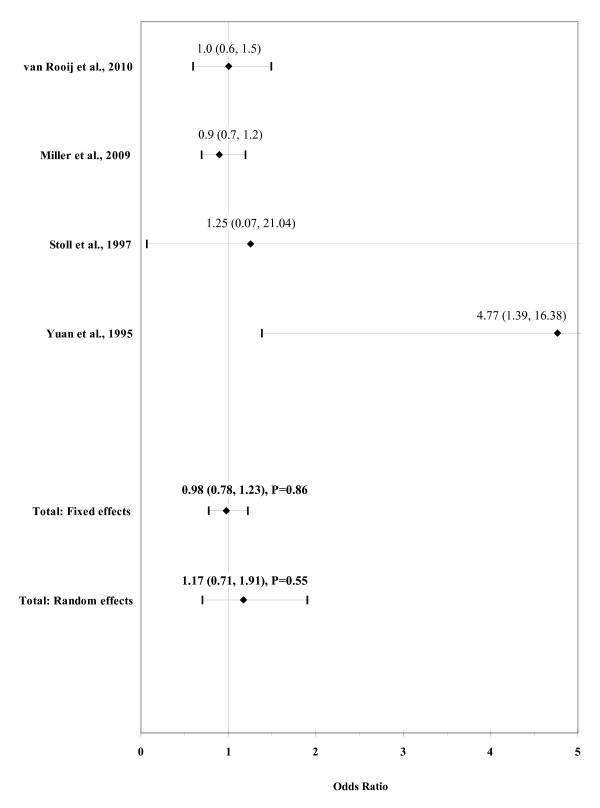
**Forest plot for maternal alcohol consumption**.

The result of the meta-analysis on the association of any maternal alcohol consumption with ARM is shown in figure 4. The I² statistic indicated heterogeneity across studies (χ² = 6.74; P = 0.08; I² = 55.5%). The estimated heterogeneity variance was tau² = 0.1172. No significant association was observed in pooled analyses using the random effects model (OR, 1.17; 95% CI, 0.71-1.91; P = 0.55). There was no evidence of publication bias (Kendall's tau = 1.36, P = 0.17; Egger's t value = 1.37, P = 0.30).

### Caffeine intake

Only the study by Miller et al. [37] reported on a potential role of caffeine exposure (table 4). Although ARM was more common among children of mothers reporting on a periconceptional use of caffeine, the association was statistically significant for the intermediate exposure group 100-299 mg only (OR, 1.9; 95% CI, 1.2-3.0).

**Table 4:** Associations between periconceptional exposures to caffeine intake

		Maternal caffeine exposure*		
			
Ref.	First author, year	Exposure	OR [95% CI]	Adjustment/matching factors
[37]	Miller, 2009	< 10 mg 10-99 mg 100-299 mg ≥300 mg	1.0 Reference 1.4 [0.9, 2.3] 1.9 [1.2, 3.0] 1.5 [0.9, 2.7]	None of the variables met the criteria for confounding by the author; therefore, only the unadjusted odds ratios were presented

* Caffeine intake per day in the year before pregnancy

### Illicit drugs

Two studies reported on the association with maternal periconceptional illicit drug use (table 5). Results were inconsistent with tentatively reduced risks in the study by van Gelder et al. [41] and increased risks in the study by Forrester and Merz [1]. Due to the small sample size, confidence intervals were very wide in both studies. Nevertheless, significantly increased risks were found by Forrester and Merz [1] for marijuana use (OR, 10.57; 95% CI, 2.87-38.96) as well as for cocaine use (OR, 6.01; 95% CI, 1.05-34.27).

**Table 5:** Associations between periconceptional exposures to illicit drugs

		Maternal illicit drug use		
			
Ref.	First author, year	Exposure	OR [95% CI]	Adjustment/matching factors
[41]	van Gelder, 2009*	Cannabis use Cocaine use	0.7 [0.4, 1.2] 0.4 [0.1, 2.7]	Adjusted for: maternal age at delivery, race or ethnicity, level of education, cigarette smoking, binge drinking, pregnancy BMI and periconceptional folic acid use
		Stimulant use	1.1 [0.3, 3.8]	Adjusted for: maternal age at delivery, level of education, binge drinking, pregnancy BMI and periconceptional folic acid use
[1]	Forrester, 2007^§†^	Methamphetamine use Cocaine use Marijuana use	3.19 [0.87, 11.73] 6.01 [1.05, 34.27] 10.57 [2.87, 38.96]	-

* Exposures at any time between one month before pregnancy and the end of the third month of pregnancy (periconceptional period).

§ Exposures during pregnancy and 1 year after delivery.

† Study reported on the ratio of the rate of illicit drug use among birth defect cases to the rate of illicit drug use among all deliveries. *We calculated the corresponding OR by data given in the article.

### Body weight

Three studies reported on the association between maternal pre-pregnancy obesity (BMI ≥30) and ARM (table 6). Risks were consistently increased in two studies (Blomberg and Källén [26]: OR, 1.87; 95% CI, 1.42-2.47; Waller et al. [40]: OR, 1.46; 95% CI, 1.10-1.95). By categorizing maternal obesity into three classes (adipositas I [BMI 30-34.9], adipositas II [BMI 35-39.9] and morbid obesity [BMI ≥40]), the closer examination by Blomberg and Källén [26] showed a particularly strong risk increase of ARM for morbid obesity (OR, 3.72; 95% CI, 1.70-7.07). Among three studies assessing maternal overweight, a significant association with ARM was observed only in van Rooij et al. [39] (OR, 1.8; 95% CI, 1.1-3.0). No such association was seen with paternal overweight in this study (OR, 0.8; 95% CI, 0.5-1.3).

**Table 6:** Associations between periconceptional exposures to body weight

		Maternal overweight/obesity		
			
Ref.	First author, year	Exposure	OR [95% CI]	Adjustment/matching factors
[26]	Blomberg, 2010	BMI < 18.5 BMI 18.5 - 24.9 BMI 25 - 29.9 BMI ≥30	1.22 [0.63, 2.13] 1.00 Reference 1.21 [0.98, 1.51] 1.87 [1.42, 2.47]	Adjusted for: maternal age, parity, smoking in early pregnancy and year of birth using the Mantel-Haenszel method
		BMI 30 - 34.9 BMI 35 - 39.9 BMI ≥40	1.77 [1.29, 2.44] 1.48 [0.74, 2.64] 3.72 [1.70, 7.07]	Adjusted for: see above
[39]	van Rooij, 2010**	BMI 25 - 29.9 BMI ≥30	1.8 [1.1, 3.0] 1.4 [0.6, 3.2]	N/A*
[40]	Waller, 2007	BMI < 18.5 BMI 25 - 29.9 BMI ≥30	0.81 [0.48, 1.36] 1.19 [0.92, 1.55] 1.46 [1.10, 1.95]	Adjusted for: maternal age, ethnicity, education, parity, smoking in the month prior to conception and supplemental folic acid intake in the month prior to conception

* The association was not confounded by any covariable.

** More detailed information on the results was obtained directly through the author.

**Figure 5:**
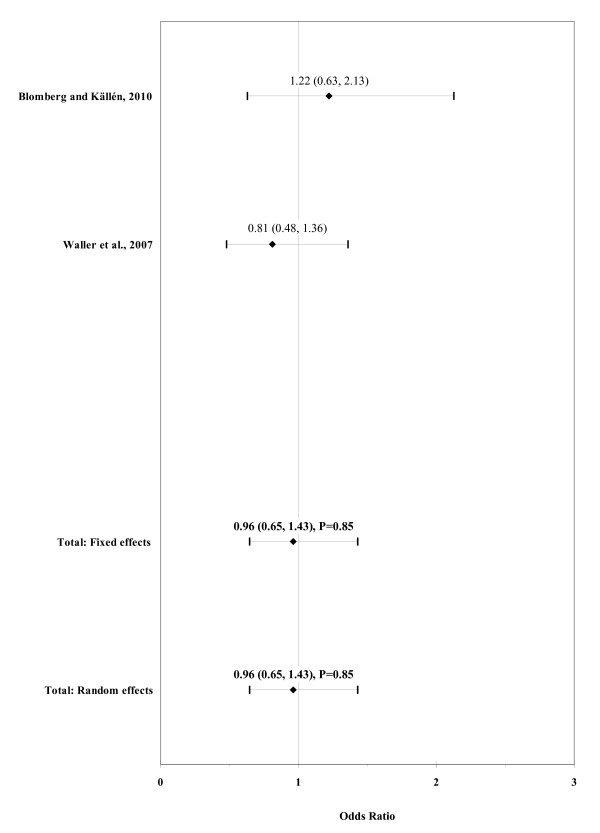
**Forest plot for maternal underweight (BMI < 18.5)**.

The result of the meta-analysis on the association of maternal underweight with ARM is shown in figure 5. The I² statistic indicated low heterogeneity across the two studies (χ² = 1.01; P = 0.32; I² = 0.5%). No significant association was observed in pooled analyses using the fixed effects model (OR, 0.96; 95% CI, 0.65-1.43; P = 0.85).

**Figure 6:**
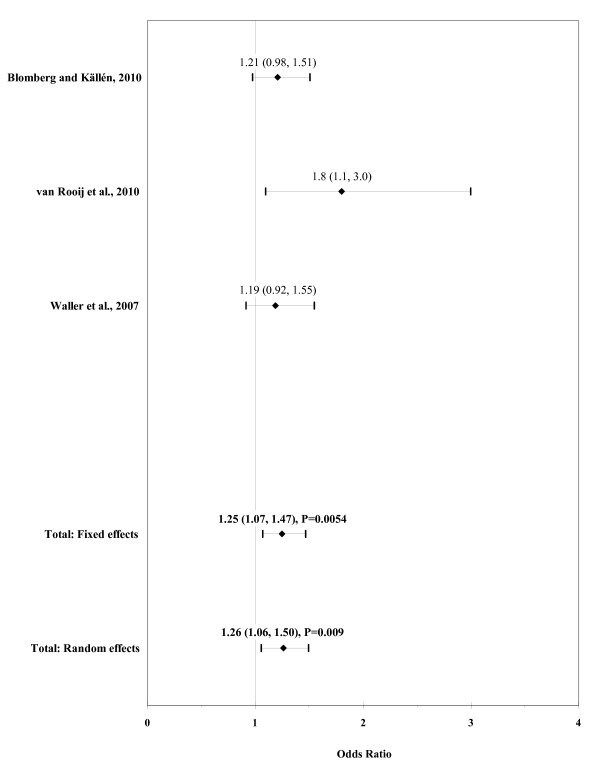
**Forest plot for maternal overweight (BMI 25-29.9)**.

The result of the meta-analysis on the association of maternal overweight with ARM is shown in figure 6. The I² statistic indicated low heterogeneity across the three studies (χ² = 2.25; P = 0.32; I² = 11.3%). In meta-analysis, a weak association was found for maternal overweight using a fixed effects model (OR, 1.25; 95% CI, 1.07-1.47; P = 0.0054). There was no evidence of publication bias (Kendall's tau = 0.52, P = 0.60; Egger's t value = 3.01, P = 0.20).

**Figure 7:**
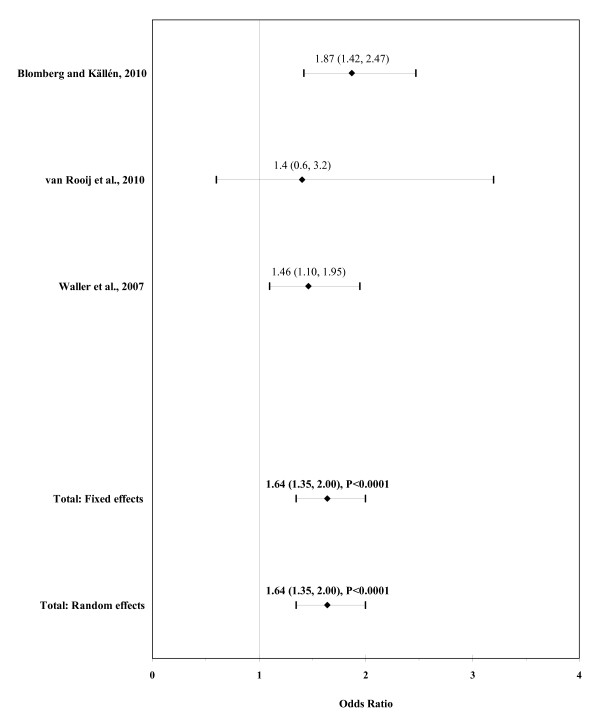
**Forest plot for maternal obesity (BMI ≥30)**.

The result of the meta-analysis on the association of maternal obesity with ARM is shown in figure 7. The I² statistic indicated homogeneity across the three studies (χ² = 1.64; P = 0.44; I² = 0%). In meta-analysis, a significant association was found for maternal obesity using a fixed effects model (OR, 1.64; 95% CI, 1.35-2.00; P < 0.0001). There was no evidence of publication bias (Kendall's tau = -0.52, P = 0.60; Egger's t value = -0.36, P = 0.78).

### Diabetes

Among eight studies assessing maternal diabetes, six differentiated between pre-existing and gestational diabetes (table 7). The closer examination of results for pre-existing diabetes showed a significantly increased risk in the study by Frías et al. [32] (OR, 2.87; 95% CI, 1.20-6.87; P = 0.04). Correa et al. [34] and Aberg et al. [24] could confirm this finding (OR, 4.70; 95% CI, 1.55-14.26 and OR, 8.18; 95% CI, 3.86-17.34; P < 0.00001) and also observed a significant association with gestational diabetes (OR, 1.91; 95% CI, 1.02-3.56 and OR, 3.29; 95% CI, 1.63-6.63; P = 0.0008). Among four studies that reported on the association with any maternal diabetes, both studies by Correa et al. [7,34] found a significantly increased risk (OR, 2.15; 95% CI, 1.31-3.55 and OR, 4.32; 95% CI, 1.50-12.47). Due to the small sample size, confidence intervals were very wide in all eight studies.

**Table 7:** Associations between periconceptional exposures to diabetes mellitus

		Maternal diabetes mellitus			
			
Ref.	First author, year	Exposure	OR [95% CI]	*P *value	Adjustment/matching factors
[25]	Bánhidy, 2010	Gestational diabetes	2.2 [0.7, 6.8]	-	Adjusted for: maternal age and employment status, birth order and maternal hypertension
[34]	Correa, 2008	Diabetes mellitus** Pre-gestational diabetes Gestational diabetes	2.15 [1.31, 3.55] 4.70 [1.55, 14.26] 1.91 [1.02, 3.56]	0.005 -	- Adjusted for: maternal age, race/ethnicity, entry into prenatal care, BMI, study center and household income
[32]	Frías, 2007*	Diabetes mellitus** Pre-gestational diabetes Gestational diabetes	1.43 [0.92, 2.25] 2.87 [1.20, 6.87] 1.18 [0.71, 1.98]	0.13 0.04 0.48	-
[24]	Aberg, 2001	Pre-gestational diabetes Gestational diabetes	8.18 [3.86, 17.34] 3.29 [1.63, 6.63]	< 0.00001 0.0008	-
[7]	Correa, 2003	Diabetes mellitus	4.32 [1.50, 12.47]	-	Adjusted for: infant's period of birth, maternal race, age, education, prenatal cigarette smoking and prenatal alcohol consumption
[36]	Martínez-Frías, 1998	Gestational diabetes	1.51 [0.60, 3.55] 1.56 [0.49, 4.40]^†^ 1.27 [0.20, 5.54]^‡^ 1.70 [0.67, 3.98]^#^ 4.19 [0.66, 26.74]^§^	-	-
[38]	Stoll, 1997	Diabetes mellitus	0.01 [0.02, 1.38]	-	-
[43]	Martínez-Frías, 1994*	Pre-gestational diabetes	2.57 [0.69, 9.60]	0.19	-

* Studies reported on the quotient of congenital anomaly frequencies only (frequency ratio: FR). We calculated the corresponding OR by data given in the articles.

** Studies reported on pre-gestational and gestational diabetes. We calculated the OR for any diabetes mellitus by data given in the articles.

† Restricted to: maternal age (≤ 34 years)

‡ Restricted to: maternal age (≥35 years)

# Restricted to: non-consanguineous parents

§ Restricted to: insulin treatment

**Figure 8:**
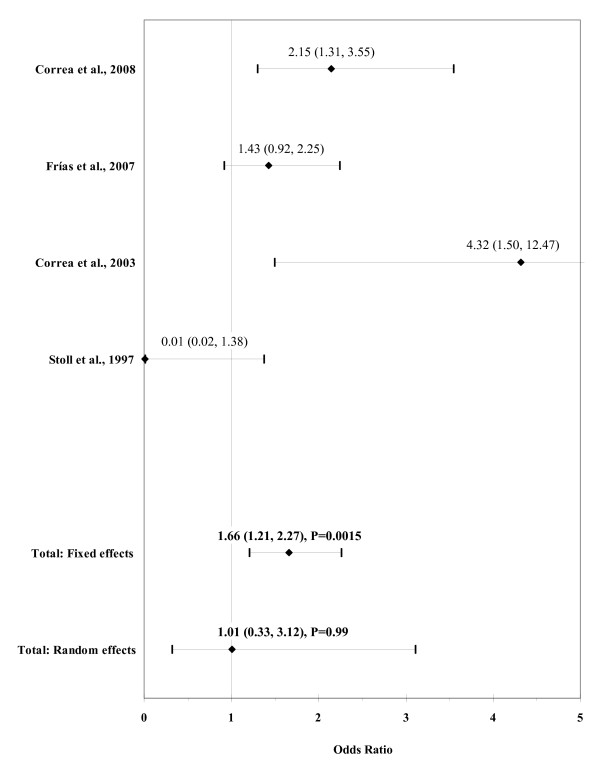
**Forest plot for any maternal diabetes mellitus**.

The result of the meta-analysis on the association of any maternal diabetes mellitus with ARM is shown in figure 8. The I² statistic indicated high heterogeneity across the four studies (χ² = 26.99; P < 0.0001; I² = 88.9%). The estimated heterogeneity variance was tau² = 1.0416. No significant association was observed in pooled analyses using the random effects model (OR, 1.01; 95% CI, 0.33-3.12; P = 0.99). There was no evidence of publication bias (Kendall's tau = 0.0, P = 1.0; Egger's t value = -0.92, P = 0.45).

**Figure 9:**
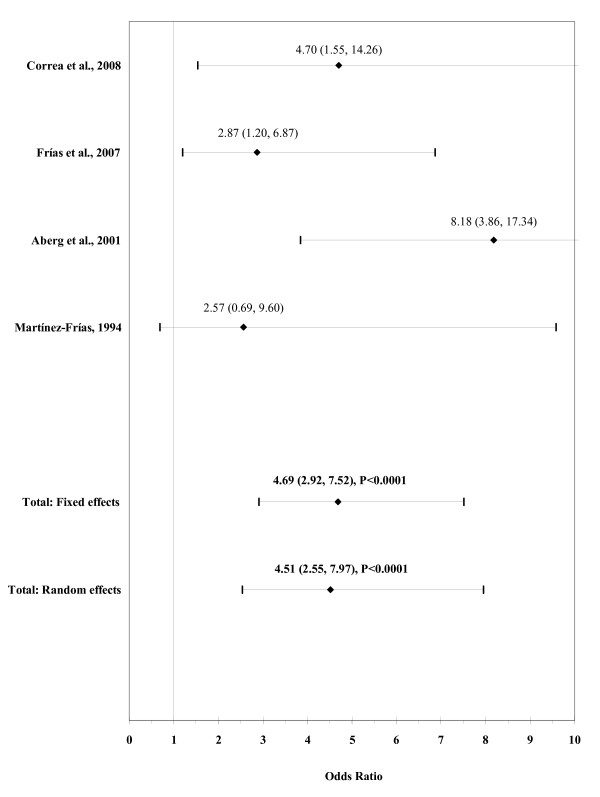
**Forest plot for maternal pre-gestational diabetes**.

The result of the meta-analysis on the association of maternal pre-gestational diabetes with ARM is shown in figure 9. The I² statistic indicated moderate heterogeneity across the four studies (χ² = 4.13; P = 0.25; I² = 27.4%). The estimated heterogeneity variance was tau² = 0.0929. A strong association was observed in pooled analyses using the random effects model (OR, 4.51; 95% CI, 2.55-7.97; P < 0.0001). There was no evidence of publication bias (Kendall's tau = -0.68, P = 0.50; Egger's t value = -1.15, P = 0.37).

**Figure 10:**
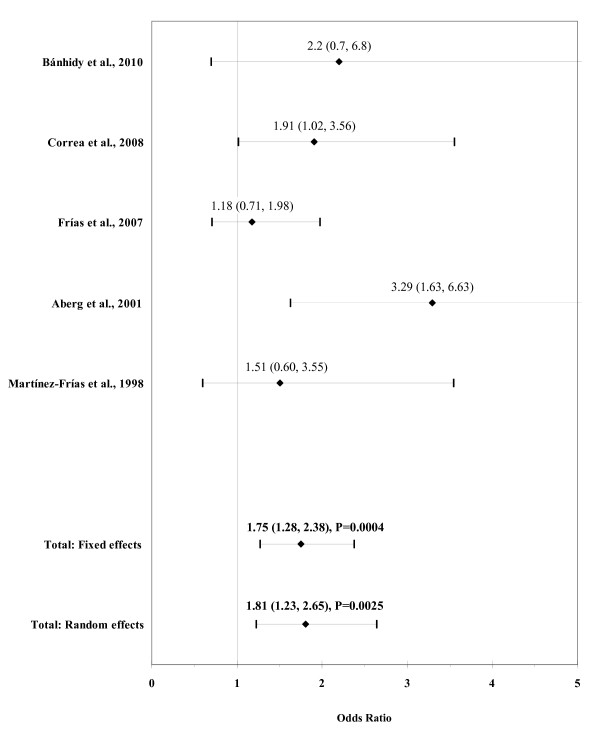
**Forest plot for maternal gestational diabetes**.

The result of the meta-analysis on the association of maternal gestational diabetes with ARM is shown in figure 10. The I² statistic indicated moderate heterogeneity across the five studies (χ² = 5.71; P = 0.22; I² = 30.0%). The estimated heterogeneity variance was tau² = 0.0570. In meta-analysis, a significant association was found for maternal gestational diabetes using a random effects model (OR, 1.81; 95% CI, 1.23-2.65; P = 0.0025). There was no evidence of publication bias (Kendall's tau = 0.98, P = 0.33; Egger's t value = 0.85, P = 0.46).

### Occupational hazard

Five studies reported on a potential role of maternal and paternal occupational hazards (table 8). Herdt-Losavio et al. [35] found a significantly increased risk with maternal janitors and cleaners (OR, 1.82; 95% CI, 1.06-3.10) and maternal scientists (OR, 2.38; 95% CI, 1.24-4.55) and Schnitzer et al. [29] with paternal vehicle manufacturers (OR, 5.1; 95% CI, 1.3-19.2). Van Rooij et al. [39] showed a suggestive association with ARM for maternal contact with industrial cleaning agents and solvents during pregnancy (OR, 2.9; 95% CI, 0.9-9.3) and for paternal contact with exhaust fumes three months before conception (OR, 1.9; 95% CI, 1.0-3.6). A significant inverse association with maternal exposure to X-ray examinations was reported in the study by Stoll et al. [38] (OR, 0.19; 95% CI, 0.09-0.38). Due to the small sample size, confidence intervals were very wide in the studies by Herdt-Losavio et al. [35], Matte et al. [28], Schnitzer et al. [29] and van Rooij et al. [39].

**Table 8:** Associations between periconceptional exposures to occupational hazard

		Maternal occupational hazard*		Paternal occupational hazard**		
				
Ref.	First author, year	Exposure	OR [95% CI]	Exposure	OR [95% CI]	Adjustment/matching factors
[39]	van Rooij, 2010	Industrial cleaning agents and solvents Cytostatics X-rays	2.9 [0.9, 9.3] 1.5 [0.3, 6.9] 0.6 [0.1, 2.6]			Adjusted for: family history of ARM and paternal smoking - Adjusted for: maternal multivitamin use
				Industrial cleaning agents and solvents Paint/varnish/adhesives/ink/thinner Welding fumes Exhaust fumes	0.6 [0.2, 1.7] 1.4 [0.6, 3.7] 1.3 [0.5, 3.3] 1.9 [1.0, 3.6]	Adjusted for: family history of ARM, maternal BMI before pregnancy, paternal smoking and paternal job exposure to exhaust fumes Adjusted for: family history of ARM Adjusted for: family history of ARM and paternal job exposure to exhaust fumes -
[35]	Herdt-Losavio, 2010	Janitors, cleaners Scientists	1.82 [1.06, 3.10]*** 2.38 [1.24, 4.55]***	-	-	Adjusted for: study centre, folic acid use, maternal age at delivery, maternal pre-pregnancy BMI, maternal race/ethnicity, maternal education, parity, maternal smoking and maternal alcohol use during the first trimester
[38]	Stoll, 1997	X-rays^†^	0.19 [0.09, 0.38]	-	-	-
[29]	Schnitzer, 1995	-	-	Carpenters, woodworkers Electricians, electrical workers	2.4 [0.7, 8.5] 1.7 [0.6, 5.0]	Matched for: race, year and hospital of birth Matched for: see above
				Printers Policemen, guards Vehicle manufacturers	2.9 [0.8, 10.2] 2.9 [0.8, 9.9] 5.1 [1.3, 19.2]	Adjusted for: maternal age and education Adjusted for: see above Adjusted for: see above
[28]	Matte, 1993	Nursing occupations	2.15 [0.83, 5.58]	-	-	-

* Job exposure during pregnancy

** Job exposure 3 months before conception

*** Job exposure 1 month prior to conception through the end of the third month of pregnancy

† Exposure to X-ray examinations

## Discussion

This systematic review and meta-analysis summarized the results of 22 studies on the association between prenatal environmental risk factors and infants born with an anorectal malformation reported between 1981 and June 2010. The majority of the studies were conducted in the United States. Case numbers ranged from 14 ARM cases in the study by Shiono et al. [33] to 564 ARM cases in the study by Honein et al. [31]. Studies were also heterogeneous with respect to control selection and adjustment for covariates. Meta-analysis was done for risk factors reported on in at least two studies, i.e. maternal and paternal smoking, maternal alcohol consumption, underweight, overweight, obesity, any maternal diabetes mellitus, pre-gestational and gestational diabetes. Consistently increased risks were observed for paternal smoking, maternal overweight, obesity and diabetes, but not for maternal smoking and alcohol consumption.

There is a great discrepancy in the reported results on the associations between maternal illicit drug use and ARM which impede comparability. Closer examination of the studies suggests that different data collection could lead to these different results. In the study by van Gelder et al. [41], mothers were interviewed by telephone by trained interviewers using a standardized questionnaire. Forrester and Merz [1] used register-based data from the Hawaii Birth Defects Program where trained staff collected information on cases through review of medical records. Thus, only consumption that had to be severe enough to be recorded in routine medical records was ascertained. It appears conceivable that illicit drug use might have been asked for and recorded more often among mothers of children with malformations than among other mothers. The discrepancy found on the associations between maternal exposures to X-rays may likewise be partly resulting from different exposure definitions. Van Rooij et al. [39] examined the maternal occupational hazard to X-rays during pregnancy whereas Stoll et al. [38] assessed women's own X-ray examinations during pregnancy.

When available, data on ARM infants with isolated anomalies (no additional major defects) were preferred in this review to data on ARM infants with multiple defects. Only six of the 22 reviewed studies looked at both groups [7,30,34,37,38,40]. Analyses, however, showed nearly the same results. Furthermore, two studies by Martínez-Frías et al. [44] and Sharpe et al. [45] were excluded because they grouped ARM with other congenital malformations (among others intestinal and tracheo-esophageal atresias) which might mix or dilute potential effects in case of diverse etiologies. The excluded studies did not find an association with the examined risk factors. In contrast, although Honein et al. [31] reported on the examination of "rectal atresia", a very rare subgroup of ARM, we included this study because it appears that the term of "rectal atresia" was used synonymously for ARM, given that the reported sample size, collected within one year, appear too high for rectal atresia and Honein et al. [31] compared their results with other studies reported on ARM. In general, there is no unique terminology for ARM that is used in the literature. Besides anorectal malformation, terms of anal atresia, anorectal atresia and imperforate anus can be found for this anomaly. Even ARM itself is a mixed group with isolated and associated malformations ranging from lower to higher forms with different genetic background [46].

Looking at some other gastro-intestinal malformations, maternal diabetes also seems to be a risk factor for esophageal atresia [25,32,34]. However, no clear association was found with gastroschisis, omphalocele, small-intestinal atresia and duodenal atresia [32,34,36,43]. There is a suggestive association between maternal overweight and omphalocele [40], but not for the other defects. Maternal obesity also seems to be a risk factor for omphalocele, whereas an inverse association was found for gastroschisis [47,48]. The use of illicit drugs including cocaine, methamphetamines and marijuana during pregnancy was found to be associated with increased risk of gastroschisis [47] and the use of cocaine, amphetamines, decongestants and pseudoephedrine was associated with intestinal atresia [49-51]. Consistently increased risks for maternal smoking were only observed for pyloric stenosis [52]. For omphalocele and gastroschisis, no consistently increased risks were found regarding alcohol consumption [47].

The significant associations with ARM and some other gastro-intestinal malformations show that the rise in maternal overweight and obesity, as well as diabetes during the last decades are of relevance for these birth defects. For example, the prevalence of overweight (obesity) in adult females from the Netherlands increased from 30% (6%) in 1981 to 42% (12%) in 2004 [53]. In girls, overweight (obesity) prevalence doubled or even tripled from 1980 to 1997 and again from 1997 to 2002-2004. The same trend was observed in the United States where 17.9% of all school-age girls were overweighed (11.7% obese) in 1999-2000 and 22.3% were overweighed (13.6% obese) in 2003-2004 [54]. During the same time, the number of women with diabetes was also increasing. The worldwide prevalence of type 1 and type 2 diabetes in women has doubled since the 1980s [55,56]. The overall prevalence was estimated to be 2.2% worldwide in 1995 and is expected to be 2.8% in 2025 with a higher proportion in the developed countries than in the developing countries (3.6% vs. 1.7% in 1995; 4.5% vs. 2.5% in 2025) [57]. Previous studies underline the need of substantial efforts to limit the obesity epidemic, which is also the main cause of the growing prevalence of diabetes among women in child-bearing age [58,59]. Besides many other beneficial health effects, such efforts could substantially reduce the risk of ARM and other birth defects in the offspring. Although associations of maternal smoking and alcohol consumption with ARM do not seem to be established based on existing evidence, the adverse health effects of these habits on the embryonic development underline the importance of avoiding them throughout pregnancies and beyond.

Our review has a number of limitations mostly resulting from the overall scarcity of published evidence. First, our meta-analysis was limited by the data provided in the individual studies. Not all studies provided risk estimates adjusted for potentially influential confounders, such as maternal age, ethnicity, education, parity, periconceptional smoking and folic acid intake [40] or maternal age, race/ethnicity, education, smoking, binge drinking, pregnancy BMI and periconceptional folic acid use [41]. Analyses of diabetes were only adjusted for BMI in the study by Correa et al. [34], known as potential influential confounder. Due to the small number of studies, we decided to pool adjusted and crude values for meta-analyses. Second, some studies used affected (malformed) control groups. Other studies used mixed controls of live-born malformed and healthy babies. A potential advantage of using malformed controls is potential reduction of response bias or recall bias that may occur when a non-malformed control group is used. On the other hand, observed associations may be biased if the risk factors of interest are also associated with the malformations of controls. Third, most sample sizes were small, so the power to detect associations was low. Fourth, despite the lack of indication of major publication bias, it is impossible to be ruled out completely, especially in the light of the low number of studies. Finally, although we searched in four databases (PubMed, EMBASE, ISI Web of Knowledge and the Cochrane library) and completed our search by reviewing related and cross-referencing literature, existence of relevant missing studies cannot be excluded.

To our knowledge, our article is the first systematic review and meta-analysis that provides an overview of the few available studies that reported on the association between prenatal environmental risk factors of the parents and ARM. Adequate evidence is still very limited. Therefore, further multicenter or register-based studies are needed to clarify the role of key risk factors for the development of ARM. One example is the recent establishment of the German Network for Congenital Uro-REctal Malformations (CURE-Net). The aim of this consortium is to collect data of affected newborns as well as of older patients with an anorectal malformation (ARM) or an exstrophy-epispadias complex (EEC) that allow to investigate molecular causes, clinical implications and psychosocial outcomes. For a standardized description of diagnostic subgroups, international classifications are used (Krickenbeck for ARM [60] and Gearhart & Jeffs for EEC [61]). Associated malformations are collected with means of the London Dysmorphology Database [62] and environmental risk factors according to the core dataset of surveillance of congenital anomalies in Europe (EUROCAT) [63]. Nationwide data acquisition should enable to achieve a sample size that is large enough to clarify the role of key risk factors for the development for ARM and EEC in general but also for each subgroup separately. Furthermore, activities are ongoing aiming to expand such a register on an international level. The recently established International Consortium on Anorectal Malformations (ARM-Net), a European collaboration between France, Italy, Germany and the Netherlands, aims to identify genetic and environmental risk factors by data sharing and combined research activities [64]. Both consortia offer the unique opportunity to establish a basis for future research to overcome current scarcity of evidence in the field of ARM, especially from populations outside the United States.

## Competing interests

The authors declare that they have no competing interests.

## Authors' contributions

Conception and design was done by HB. Literature review, data extraction and statistical analysis were carried out by NZ and EJ. Drafting of the article was done by NZ. Revision of the article was done by NZ, EJ and HB. All authors read and approved the final manuscript.
